# Oxidation of ferumoxytol by ionizing radiation releases iron. An electron paramagnetic resonance study

**DOI:** 10.1093/jrr/rrac008

**Published:** 2022-03-17

**Authors:** Michael S Petronek, Douglas R Spitz, Garry R Buettner, Bryan G Allen

**Affiliations:** Department of Radiation Oncology, Free Radical and Radiation Biology, The University of Iowa, Iowa City, IA 52242-1181, USA; Department of Radiation Oncology, Free Radical and Radiation Biology, The University of Iowa, Iowa City, IA 52242-1181, USA; Department of Radiation Oncology, Free Radical and Radiation Biology, The University of Iowa, Iowa City, IA 52242-1181, USA; Department of Radiation Oncology, Free Radical and Radiation Biology, The University of Iowa, Iowa City, IA 52242-1181, USA

**Keywords:** ferumoxytol (FMX), magnetite, redox chemistry, Fricke dosimetry, iron oxide radiolysis, EPR spectroscopy

## Abstract

Ferumoxytol (FMX) is an iron oxide nanoparticle that is FDA approved for the treatment of iron deficiency anemia. FMX contains an Fe_3_O_4_ core. Currently, the redox chemistry of Fe_3_O_4_ nanoparticles remains relatively unexplored. FMX has recently gained interest as an anti-cancer agent. Ionizing radiation (IR) is a treatment modality employed to treat several types of cancer. Utilizing electron paramagnetic resonance (EPR) spectroscopy, we found that the products produced from the radiolysis of water can oxidize the Fe_3_O_4_ core of FMX. Because of the limited diffusion of the HO_2_^•^ and HO^•^ produced, these highly oxidizing species have little direct effect on FMX oxidation. We have determined that H_2_O_2_ is the primary oxidant of FMX. In the presence of labile Fe^2+^, we found that reducing species generated from the radiolysis of H_2_O are able to reduce the Fe^3+^ sites of the Fe_3_O_4_ core. Importantly, we also have shown that IR stimulates the release of ferric iron from FMX. Because of its release of iron, FMX may serve as an adjuvant to enhance radiotherapy.

## INTRODUCTION

Ferumoxytol (Feraheme®, FMX) is an FDA-approved therapeutic for the treatment of iron-deficiency anemia [1, 2]. FMX is a 30 nm, neutral charged superparamagnetic iron oxide nanoparticle (SPION) with an Fe_3_O_4_ core (formally, 2Fe^3+^,1Fe^2+^ oxide) encapsulated within a carboxylated polymer coating [3]. In addition to iron deficiency anemia, FMX has also been utilized as a magnetic resonance imaging (MRI) contrast agent in the imaging of glioma tumors in patients that are unable to receive gadolinium [4–6]. Recently, FMX has shown promise as an anti-cancer agent [7]. It has been shown to enhance leukemia cell killing in cells with low ferroportin expression. These observations suggest the hypothesis that FMX-induced cell death may potentially be an iron- and reactive oxygen species (ROS)-dependent process. Thus, the Fe_3_O_4_ core of FMX may be redox-active, thereby providing the iron needed for detrimental oxidations.

Ionizing radiation (IR) is a common cancer therapeutic used as a treatment modality in a variety of cancer types. Recent literature suggests that SPIONs may function as radiosensitizers, by increasing DNA damage via enhanced production of ROS [8]. IR readily leads to the oxidation of Fe^2+^ to Fe^3+^; because iron can enhance cellular free radical oxidation reactions [9, 10], approaches that increase redox active iron in cancer cells may increase radiosensitization.

The oxidation of ferrous iron following the radiolysis of H_2_O (Fricke dosimetry), is a widely accepted dosimetric technique that has been utilized since 1927 [11]. Fricke dosimetry allows for IR dose estimation to water (D_w_) by evaluating changes in optical density (OD) associated with the oxidation of ferrous (Fe^2+^) to ferric (Fe^3+^) iron [12, 13]. For a given D_w_, the radiolytic yield of Fe^3+^ (G (Fe^3+^)) can be approximated by measuring changes in OD (alias absorbance) (equation [1]) [12, 14]):

(1) \begin{equation*} G\left({\mathbf{Fe}}^{\mathbf{3}+}\right)=\frac{\Delta \mathbf{OD}\ }{{\mathbf{D}}_{\mathbf{w}}\ast \boldsymbol{\varepsilon} \ast \mathbf{d}\ast \boldsymbol{\rho}}\ \left(\mathbf{mol}\ {\mathbf{J}}^{-\mathbf{1}}\right) \end{equation*}

where (G (Fe^3+^) is defined as the number of Fe^3+^ ions produced per 100 eV of energy deposition, ε is the molar extinction coefficient (here the extinction coefficient would be that of Fe^3+^ at 303 nm minus the extinction coefficient of Fe^2+^ at 303 nm, 2174 M^−1^ cm^−1^ [11]), d is the absorption pathlength and ρ is the density of the solution (taken as 1.00 g cm^−3^).

A major focus of this project was to develop a reliable method to detect FMX and determine its concentration in water-based solutions. We hypothesized that electron paramagnetic resonance (EPR) spectroscopy would be an ideal approach because there are two low-spin (S = 1/2) Fe^3+^ in each Fe_3_O_4_ with minimal contribution from the low-spin Fe^2+^ (S = 0) allowing for oxidation state specificity. In addition, EPR would provide a useful tool to evaluate levels of FMX in complex environments such as cell culture media, blood, or tissue. Our goal is to apply these principles to understand the radiation chemistry of Fe_3_O_4_ nanoparticles to evaluate any chemical changes upon exposure to IR.

## MATERIALS AND METHODS

### Chemical preparations

FMX (Feraheme®) was diluted to the appropriate concentration in 18 MΩ H_2_O. For pH dependency experiments, 50 μM FMX in 18 MΩ H_2_O was titrated to the appropriate pH with either 1 M HCl or 1 M NaOH. For mechanistic experiments, FMX was diluted to 50 μM in 18 MΩ H_2_O supplemented with either 50 mM Na-pyruvate, D-mannitol, or ferrous ammonium sulfate (FAS). Na-pyruvate (ThermoFisher Scientific; 11 360 070), D-mannitol, and FAS were diluted to 50 mM from a 100 mM stock. Samples were irradiated with a ^60^Co source.

### EPR spectroscopic evaluation of FMX

FMX concentrations were determined by measuring the peak-to-peak signal intensity of the EPR spectra of the low-spin Fe_3_O_4_ complex at *g* = 2 relative to a standard curve. The following scan parameters were used: center field = 3508.97 G, sweep rate = 2000 G/42 s, time constant = 327.68 ms, frequency = 9.85 GHz, power attenuation = 18 dB, modulation frequency = 100 kHz, modulation amplitude = 0.7 G, with spectra being generated from a signal average of 2 scans. The Fe^3+^ concentration of FMX was calculated based on a 2:1 stoichiometry of 2Fe^3+^:Fe^2+^ contained within a magnetite crystal and a FMX molecular weight of 731 kDa [15]. Samples were examined by EPR within 10 min of irradiation.

### Detection of Fe release

Detection of Fe-release from FMX was accomplished by diluting the appropriate FMX samples into a ferrozine buffer (5 mM ferrozine diluted in double-distilled H_2_O) ± 5 mM ascorbate. The formation of the Fe^2+^-ferrozine complex, absorption at 562 nm (ε_562_ = 27.9 mM^−1^ cm^−1^) [16], was evaluated using a Beckman DU800 UV–Vis spectrometer. Ferrozine buffer containing 5 mM ascorbate was used to reduce all the chelatable iron to Fe^2+^ ([Fe]_total_). The amount of Fe^3+^ released was calculated as the difference between [Fe]_total_ (ferrozine +5 mM ascorbate) and [Fe^2+^] (ferrozine alone) (equation [2**]**):

(2) \begin{equation*} \left[\mathrm{Fe}^{3+}\right]\ \left(\mathrm{nM}\right)=\left[\mathrm{Fe}\right]_\mathrm{total}\hbox{--} \left[\mathrm{Fe}^{2+}\right]\ \left(\mathrm{nM}\right) \end{equation*}

## RESULTS AND DISCUSSION

### FMX is readily detected using EPR spectroscopy

**Fig. 1.**
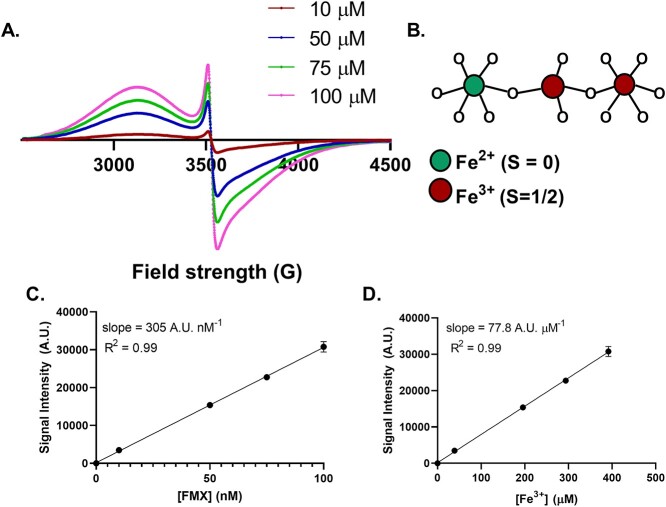
FMX is readily detected with EPR spectroscopy in room temperature aqueous solution. (A) Increasing concentrations of FMX in 18 MΩ H_2_O, pH ≈ 7 were examined using an X-band Bruker EMX spectrometer. (B) Fe_3_O_4_ structure with low-spin, octahedral Fe^2+^and Fe^3+^ and a central, tetrahedral Fe^3+^. (C) FMX concentration-dependence was determined by measuring the peak-to-peak signal intensity at *g ≈ 2* (*≈* 3500 G). (D) [Fe^3+^] concentration dependence was performed by approximating the FMX Fe^3+^ content and comparing to the peak-to-peak signal intensity at *g ≈ 2* (*≈* 3500 G).

The goal of this project was to develop a reliable method to detect FMX and determine its concentration in water-based solutions. We hypothesized that EPR spectroscopy would be an ideal approach because there are two low-spin (S = 1/2) Fe^3+^ in each Fe_3_O_4_ with minimal contribution from the low-spin Fe^2+^ (S = 0) allowing for oxidation state specificity. In addition, EPR would provide a useful tool to evaluate levels of FMX in complex environments such as cell culture medium, blood and tissue. Using EPR, we detected an Fe_3_O_4_ concentration-dependent-signal at *g* ≈ 2 (detected at ≈ 3500 G) with a second absorption at *g* ≈ 2.3 (detected at ≈3100 G) (Fig. 1A) [17]. This suggests detection of the low-spin (S = 1/2) Fe^3+^ contained within the octahedral sublattice (*g* = 2) along with the tetrahedral lattice (*g*_‖_ = 2, *g*_⊥_ = 2.3) of the magnetite structure (Fig. 1B) [18]. We quantified the peak-to-peak intensity of the signal and verified that it had a direct linear dependence on concentration (Fig. 1C). Only Fe^3+^ would contribute to this signal because of the low-spin nature of the crystal structure; low-spin Fe^2+^ (S = 0) is EPR silent. Because Fe_3_O_4_ has a 2:1 Fe^3+^:Fe^2+^ stoichiometry, we could approximate the Fe^3+^ content and its linear proportionality with the *g* ≈ 2 peak-to-peak signal intensity (Fig. 1D). The peak at *g* ≈ 2 has contributions from both the tetrahedral lattice and octahedral sublattice, thus acting as a more robust marker of the total Fe^3+^ content [18]. Therefore, the EPR spectroscopic method should provide an accurate measure of the Fe^3+^ content in FMX.

### FMX undergoes IR-induced oxidation

Traditional Fricke dosimetry is performed by detecting changes in OD following the oxidation of Fe^2+^ to Fe^3+^ by the products of the radiolysis of water [13]. However, because the EPR signal intensity at *g* ≈ 2 is linearly proportional to both FMX and the content of Fe^3+^ in FMX, EPR signal intensity can also be used. We hypothesized that FMX can undergo Fricke-type chemical reactions leading to the oxidation of its Fe^2+^ sites and these changes could be detected by EPR. The approximate radiolytic yield (G(Fe^3+^)) would be given by equation (3):

(3) \begin{equation*} \mathbf{G}\left({\mathbf{Fe}}^{\mathbf{3}+}\right)=\frac{\mathbf{\Delta }\mathbf{SI}\ }{{\mathbf{D}}_{\mathbf{w}}\ast \frac{\mathbf{d}\mathbf{SI}}{\mathbf{d}\left[{\mathbf{Fe}}^{\mathbf{3}+}\right]}\ast{\mathbf{MW}}_{\mathbf{Fe}}}\ \left(\mathbf{mmol}\ {\mathbf{J}}^{-\mathbf{1}}\right) \end{equation*}

where ΔSI is the change in EPR signal intensity at *g* ≈ 2, D_w_ is the dose of IR to water, dSI/d[Fe^3+^] is the change in signal intensity per μM Fe^3+^ = 77.8 A.U. per μM Fe^3+^ (Fig. 1C), and MW_Fe_ is the molecular weight of Fe (55.84 g mol^−1^). (Note: 77.8 A.U. per μM Fe^3+^ is specific to the physical setup and instrument settings of these specific experiments.)

**Fig. 2.**
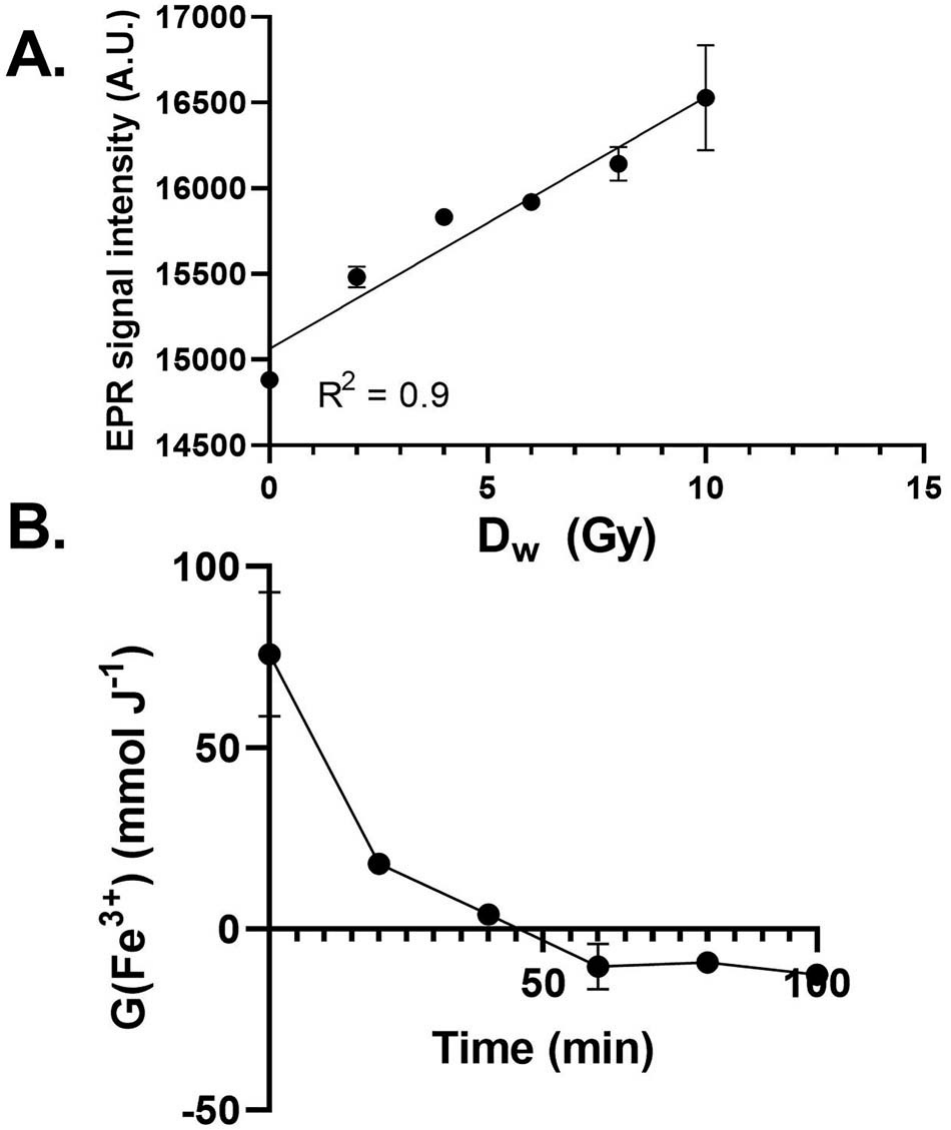
FMX undergoes IR-induced oxidation. (A) 50 nM FMX in 18 MΩ H_2_O was irradiated at increasing doses (0–10 Gy) using a ^60^Co source at 0.6 Gy min^−1^. EPR spectroscopic evaluation of the peak-to-peak signal intensity at *g ≈ 2* (*≈* 3500 G) was done within 10 min of sample irradiation. (B) Temporal dependence of IR-induced oxidation of FMX. 50 nM FMX in 18 MΩ H_2_O was irradiated with 10 Gy (0.6 Gy min^−1^) using a ^60^Co source. EPR spectroscopic evaluation of the peak-to-peak signal intensity at *g ≈ 2* (*≈* 3500 G) was done within 10 min of sample irradiation and then every 20 min up to 100 min following irradiation.

To determine if Fe_3_O_4_ could undergo ionization following the radiolysis of water, 50 μM FMX in double-distilled H_2_O was irradiated with increasing doses (D_w_). We observed an increase in EPR signal intensity with increasing doses of IR (Fig. 2A). This suggests that IR may lead to the oxidation of the Fe^2+^ sites within the cluster; the increase in EPR signal intensity is consistent with equation (3) for doses <10 Gy. Thus, FMX oxidation is also consistent with traditional Fricke dosimetric measures of FeSO_4_ at clinically relevant doses that show a linear proportionality between OD changes and D_w_ [13].

To determine if there was any long-term temporal dependence associated with FMX oxidation following radiation, G(Fe^3+^) was calculated at multiple time points (Fig. 2B). We found that following the initial radiolytic oxidation of FMX, there was a steady decline in radiolytic yield overtime. After 60 min, G(Fe^3+^) becomes negative and then remains stable for up to 100 min. This suggests that the initial oxidation event stimulates the decomposition of FMX that continues until all the oxidized surface charges have been removed, leaving behind a slightly smaller Fe_3_O_4_ core.

**Fig. 3.**
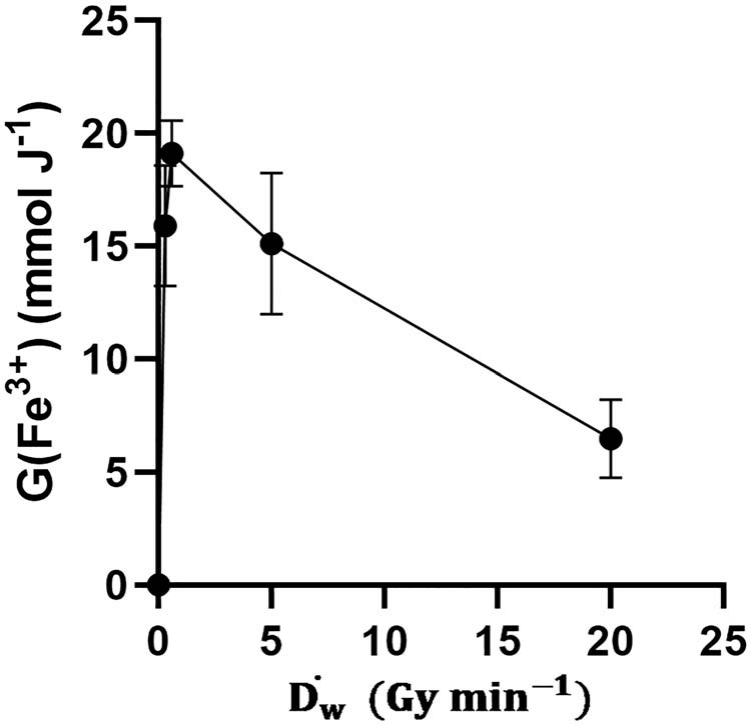
FMX oxidation is dose rate dependent. 50 nM FMX in 18 MΩ H_2_O was irradiated at increasing dose rates (10 Gy) using a ^60^Co and altering the source to sample distance. EPR spectroscopic evaluation of the peak-to-peak signal intensity at *g ≈ 2* (3500 G) was done within 10 min of sample irradiation.

Next, we determined if the oxidation of Fe_3_O_4_ was dependent on the dose-rate of the IR. We found that FMX oxidation reaches a maximum at 0.6 Gy min^−1^ (Fig. 3). This is consistent with a dose rate-dependent suppression of G(Fe^3+^) at a dose rates <100 Gy s^−1^ with a monoenergetic beam [19]. O’Leary *et al.* proposed that this effect is the result of recombination of free radicals following the radiolysis of water at high dose rates. Our data support this notion, but it may be further compounded due to the diffusion limitations of the crystal core, as Fe_3_O_4_ oxidation is limited by the rate of diffusion of O_2_ into the core [20]. Thus, lower IR dose rates likely enhance Fe_3_O_4_ oxidation by providing a steady flow of oxidation reactions over a longer period of time, thereby increasing the probability of diffusion of O_2_ into the Fe_3_O_4_ core and lowering the probability of recombination events.

### FMX undergoes non-traditional Fe radiochemistry

We then determined the main drivers of FMX oxidation by the radiolysis of water. Classically, the radiolytic yield of Fe^3+^ in the Fricke system upon the radiolysis of water is described by (equation [4]) [21]:

(4) \begin{equation*} \left(\mathbf{Fe}^\mathbf{3+}\right)=\mathbf{3}\mathbf{G}\left(\mathbf{H}^\bullet \right)+\mathbf{2}\mathbf{G}\left(\mathbf{H}_\mathbf{2}\mathbf{O}_\mathbf{2}\right)+\mathbf{G}\left(\mathbf{H}\mathbf{O}^\bullet \right)+\mathbf{3}\mathbf{G}\left(\mathbf{H}\mathbf{O}_\mathbf{2}^\bullet \right)\mathbf{G} \end{equation*}

The radiolysis of H_2_O, under our experimental system results in many different reactive species, including e^−^_aq_, H^•^, HO^**•**^, O_2_^•-^ and its conjugate acid HO_2_^•^, as well as H_2_O_2_ and other products in small yields.

The deposition of energy into water can result in homolytic bond cleavage, yielding H^•^ and HO^•^:

(5) \begin{equation*} \mathrm{H}2\mathrm{O}^\ast \to \mathrm{H}^\bullet +\mathrm{HO}^\bullet \end{equation*}

Or this energy can ionize water:

(6) \begin{equation*} \mathrm{H}2\mathrm{O}+\mathrm{IR}\to \mathrm{H}_2\mathrm{O}^\bullet ++\mathrm{e}-\mathrm{aq} \end{equation*}

then:

(7) \begin{equation*} \mathrm{e}-\mathrm{aq}+\mathrm{H}+\mathrm{aq}\to \mathrm{H}^\bullet \end{equation*}

and:

(8) \begin{equation*} \mathrm{H}2\mathrm{O}^\bullet +\rightarrow \mathrm{H}+\mathrm{aq}+\mathrm{HO}^\bullet \end{equation*}

Both e^−^_aq_ and H^•^ wiil rapidly react with O_2_ to form superoxide or its conjugate acid, the hydroperoxyl radical:

(9) \begin{equation*} \mathrm{O}2+\mathrm{e}-\mathrm{aq}\to \mathrm{O}_2^{\bullet -} \end{equation*}

(10) \begin{equation*} \mathrm{O}2+\mathrm{H}^\bullet \to \mathrm{HO}_2^\bullet \left(\mathrm{p}K\mathrm{a}\ \left(\mathrm{HO}_2^\bullet \right)=4.8\right) \end{equation*}

HO_2_^•^can then efficiently oxidize Fe^2+^ (equation [11]):

(11) \begin{equation*} \mathrm{H}\mathrm{O}_2^\bullet +\mathrm{Fe}^{2+}+\mathrm{H}^+\to \mathrm{H}_2\mathrm{O}_2+\mathrm{Fe}^{3+} \end{equation*}

**Fig. 4.**
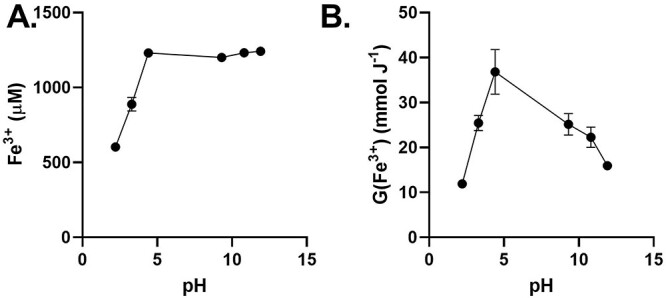
The effects of the radiolysis of water on FMX is pH dependent. (A) 50 nM FMX in 18 MΩ H_2_O with variable pH (titrated with either 1 M HCl or 1 M NaOH) and then was irradiated with 10 Gy using a ^60^Co source at 0.6 Gy min^−1^. (B) EPR spectroscopic evaluation of the peak-to-peak signal intensity at *g ≈ 2* (*≈* 3500 G) was done within 10 min of sample irradiation to determine the radiolytic yield, G(Fe^3+^). Results of triplicate measures ± SD.

Because the fraction of HO_2_^•^ present of the O_2_^•-^/HO_2_^•^ dyad is pH-dependent, we examined whether the oxidation of FMX is also pH-dependent. We found that at low pH there is a decrease in FMX signal intensity indicative of a decrease in Fe^3+^ (Fig. 4A). This is consistent with the low-temperature reduction of Fe_3_O_4_ under acidic conditions (< 500°C) [22, 23]. We found that maximal FMX oxidation occurred following 10 Gy IR at pH = 5; oxidation was reduced at both lower and higher pH ranges (Fig. 4B). This suggests that HO_2_^•^ may not play a critical role in FMX radiochemistry because the HO_2_^•^ population increases under more acidic conditions (p*K*_a_ = 4.8) and typically functions as an oxidizing species to increase G(Fe^3+^) (equation [4]) [24, 25].

Next, we determined whether H_2_O_2_ or HO^•^ has a greater effect on the oxidation of FMX by IR. Ionization of H_2_O by radiation leads to H_2_O_2_  *via* the hydroperoxyl radical (equation [11]). The generation of H_2_O_2_ can oxidize two Fe^2+^ ions *via* Fenton chemistry. This first oxidation occurs *via* Fenton chemistry directly (equation [12]):

(12) \begin{equation*} \mathrm{H}_2\mathrm{O}_2+\mathrm{Fe}_2+\to \mathrm{Fe}^{3+}+\mathrm{OH}^-+\mathrm{HO}^\bullet \end{equation*}

The second oxidation occurs indirectly, i.e. by the HO^•^ produced from Fenton chemistry (equation [13]):

(13) \begin{equation*} \mathrm{HO}^\bullet +\mathrm{Fe}^{2+}\to \mathrm{OH}^-+\mathrm{Fe}^{3+} \end{equation*}

**Fig. 5.**
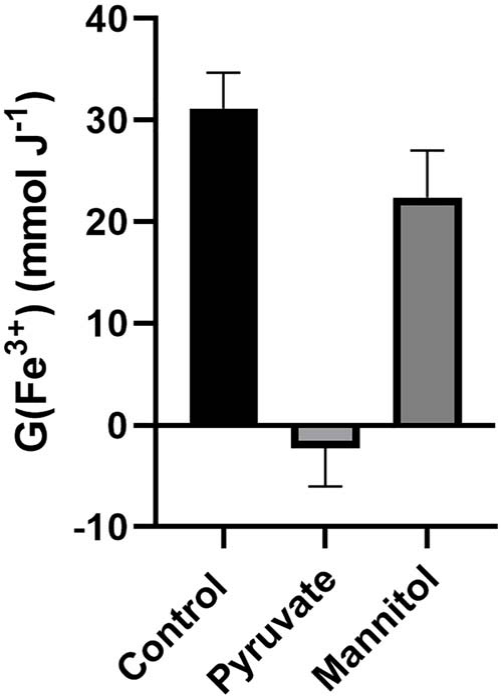
Oxidation of FMX is primarily dependent on H_2_O_2_. Control: Irradiation of 50 nM FMX in18 MΩ H_2_O with 10 Gy using a ^60^Co source at 0.6 Gy min^−1^ yields G(Fe^3+^) = 31 mol J^−1^ (using equation [3]). When this solution of FMX contains with 50 mM Na-pyruvate the apparent G(Fe^3+^) is essentially 0. 50 mM D-mannitol decreased the apparent G(Fe^3+^) by about 30% of control. EPR spectroscopic evaluation of the peak-to-peak signal intensity at *g ≈ 2* (*≈* 3500 G) was done within 10 min of sample irradiation. Results of triplicate measures ± SD.

The same oxidation of Fe^2+^ by HO^•^ may occur directly from IR as HO^•^ is one of the oxidants produced by the radiolysis of H_2_O (equations [5] and [8]**)**. To examine this possibility, FMX was placed in H_2_O supplemented with either 50 mM pyruvate, to act as an Fe-independent H_2_O_2_ scavenger, or 50 mM mannitol, to scavenge HO^•^ [26–28]. That the addition of pyruvate lowered the apparent value of G(Fe^3+^) to essentially 0, following 10 Gy IR; that is, there is no change in signal intensity when comparing irradiated and unirradiated samples, ΔSI of equation (3) is essentially 0. Mannitol decreased G(Fe^3+^) by approximately 30%, from 31 to 22 mmol J^−1^ (Fig. 5). Because HO^•^ is highly reactive, the decrease in radiolytic yield provided by mannitol is likely the result of its reaction with HO^•^, thereby preventing site-specific reactions within the Fe_3_O_4_ core.

**Fig. 6.**
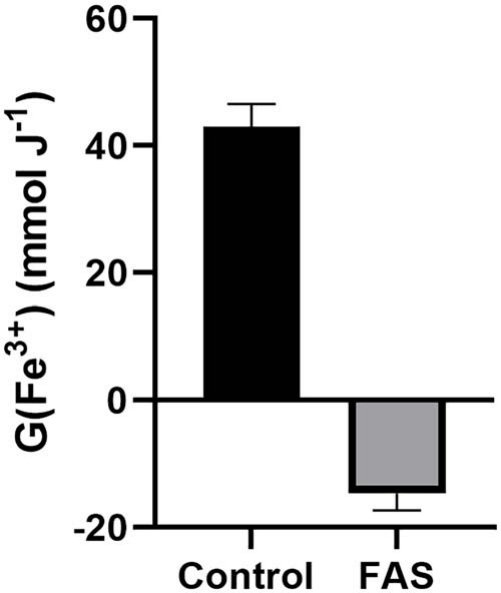
FMX can be reduced by IR in the presence of labile Fe^2+^. Control: Irradiation of 50 nM FMX in18 MΩ H_2_O with 10 Gy using a ^60^Co source at 0.6 Gy min^−1^ yields G(Fe^3+^) = 43 mol J^−1^ (using equation [3]). When this solution of FMX contains with 50 mM Fe^2+^ (FAS) the apparent G(Fe^3+^) is reduced to −15 mmol J^−^. EPR spectroscopic evaluation of the peak-to-peak signal intensity at *g ≈ 2* (*≈* 3500 G) was done within 10 min of sample irradiation. Results of triplicate measures ± SD.

Lastly, we aimed to evaluate the impact of reducing species (e.g. e^−^_aq_ and O_2_^●-^) produced following the radiolysis of H_2_O in our system. These species should be considered for the redox chemistry associated with mixed iron oxides such as Fe_3_O_4_ because reductants may affect the Fe^3+^ sites. While the radiolysis of Fe typically considers the oxidation of Fe^2+^ (equation [4]), the reduction of Fe^3+^ may be relevant chemically given the 2:1 Fe^3+^:Fe^2+^ stoichiometry of Fe_3_O_4_. To evaluate the impact of these reducing species produced, we irradiated FMX with 10 Gy in H_2_O containing 50 mM Fe^2+^ (FAS). Thus, we can leverage the rapid oxidation of Fe^2+^ to absorb HO^●^, H_2_O_2_, H^●^ and HO_2_^●^ allowing reduction chemistry to occur. We found that the addition of 50 mM Fe^2+^ to the H_2_O resulted in a 134% decrease in radiolytic yield from 43 mol J^−1^ to −15 mol J^−1^ (Fig. 6). The generation of a negative G(Fe^3+^) by the addition of labile Fe^2+^ is indicative of a site-specific reduction of the Fe^3+^ sites by reducing species produced from the radiolysis of H_2_O such as e^−^_aq_ and O_2_^●-^. These results are unsurprising as the Fe^3+^-OOH core of ferritin has been shown to be labilized by IR using a pulsed-radiolysis approach that was attributed to the e^−^_aq_ produced from the radiolysis of H_2_O [29]. Additionally, this may illustrate the potential for site-specific reactions with oxygen inside the crystal core by H^●^ further enabling Fe reduction chemistry to occur. Therefore, in the presence of labile or freely chelatable Fe^2+^ (as is seen in living systems) the reduction of the Fe^3+^ sites of FMX by radiolytically produced species such as e^−^_aq_ and O_2_^●-^ may become increasingly relevant.

**Fig. 7.**
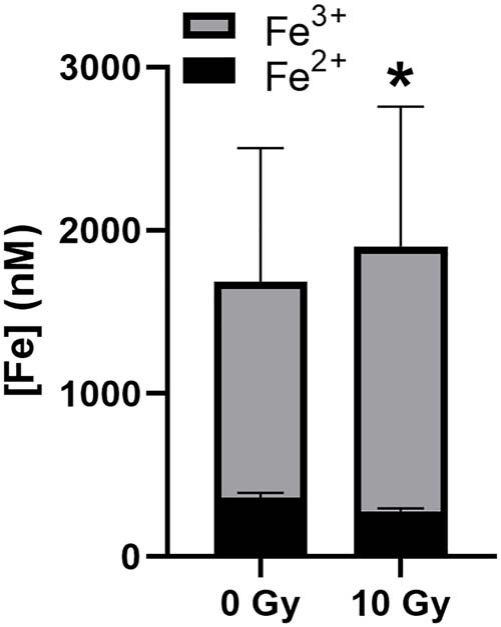
IR liberates Fe^3+^ from the FMX core. 50 nM FMX in 18 MΩ H_2_O was irradiated with 10 Gy using a ^60^Co source at 0.6 Gy min^−1^. Free Fe^2+^ was evaluated by diluting samples in 5 mM ferrozine buffer and evaluating the absorbance at 562 nm (ε_562_ = 27.9 mM^−1^ cm^−1^). Total Fe was done by diluting samples in 5 mM ferrozine with 5 mM ascorbate. Free Fe^3+^ was calculated as the difference between [Fe]_total_ and [Fe^2+^]. Results of triplicate measures ± SD. ^*^P < 0.05 using a paired, two-tailed Student’s T-test.

A key question remains: Does IR enhance Fe release from the Fe_3_O_4_ core into the supporting solvent? To address this, solutions containing 50 μM FMX in distilled water were irradiated with 10 Gy IR and then analyses for iron were employed as presented in Methods. Following IR there was a significant increase in total Fe released from FMX of 218.6 ± 54.2 nM (14.7 ± 5.4%; *P* < 0.05) (Fig. 7). As we previously detected the oxidation of the Fe^2+^ sites contained with FMX, we hypothesized that these atoms would be those likely released. Consistent with this hypothesis, we found that there was a 301.1 ± 45.1 nM (31.6 ± 19.5%) increase (*P* < 0.05) in Fe^3+^ released from the core. This result was accompanied by a 82.4 ± 10.6 nM (22.8 ± 1.4%) decrease (*P* < 0.05) in Fe^2+^ being released. These findings suggest that IR enhances the release of Fe from FMX by oxidizing the Fe^2+^ sites. Taken together, these data suggest that redox reactions associated with the radiolysis of H_2_O can enhance the release of Fe from the FMX core.

## CONCLUSION

In this study, we have made the following observations regarding FMX radiochemistry:

- EPR spectroscopy is a useful tool for evaluating FMX concentrations and Fe_3_O_4_ redox chemistry;
- IR can lead to the oxidation of FMX;
- FMX undergoes non-traditional Fe-radiochemistry as H_2_O_2_ appears to be the primary oxidant due to its ability to diffuse into the crystal core;
- HO_2_^•^ and HO^•^ likely only contribute site-specific oxidations because their chemistries are diffusion-rate limited;
- In the presence of labile Fe^2+^ (as seen in living systems), radiolytically produced species such as e^−^_aq_ and O_2_^●-^ can reduce the Fe^3+^ sites of FMX and should not be ignored;
- IR can stimulate the release of Fe^3+^ from the FMX core.

While significant pre-clinical work remains, these data suggest the possibility of FMX as a clinically relevant, redox active Fe reserve to enhance radiotherapy.
